# Utilisation of partogram at a district in the North West Province, South Africa

**DOI:** 10.4102/hsag.v29i0.2175

**Published:** 2024-01-25

**Authors:** Suzan K.M. Mabasa, Molekodi J. Matsipane, Ushotanefe Useh

**Affiliations:** 1 Department of Nursing Science, Faculty of Health Sciences, Sefako Makgatho Health Sciences University, Pretoria, South Africa; 2 Department of Nursing Science, Faculty of Health Sciences, North-West University, Mafikeng, South Africa; 3 Department of Lifestyle Diseases, Faculty of Health Sciences, North-West University, Mafikeng, South Africa

**Keywords:** partogram or partograph, utilisation, midwives, labour, healthcare facility

## Abstract

**Background:**

The partogram or partograph is a tool used to monitor the progress of labour and serves as a diagnostic tool for labour-related abnormalities such as prolonged labour, cephalopelvic disproportion (CPD) and obstructed labour. Appropriate utilisation of the partogram aids health caregivers with early diagnosis and facilitates clinical judgement and interventions to prevent complications of abnormal labour. The partogram is thus a mandatory tool to be utilised to monitor the progress of labour for intrapartum care in South Africa.

**Aim:**

This study aimed to assess and describe the utilisation of the partogram in a district of the North West Province.

**Setting:**

The study was conducted in the private rooms of facilities rendering maternity services in the district.

**Methods:**

A quantitative cross-sectional descriptive design was employed. A purposive sampling was used to select healthcare facilities, and simple random sampling was employed to select plotted partograms. Data were collected using a checklist and analysed using Statistical Package for Social Sciences software version 22.

**Results:**

A total of 279 partograms were analysed. The average partogram utilisation was 20% correct and 80% substandard or not recorded. All files had partogram documents included.

**Conclusion:**

A large percentage (80%) of the partograms were not completed according to the World Health Organization (WHO) standards. There was a concern about high proportions of unrecorded parameters such as monitoring of foetal and maternal conditions, and the progress of labour.

**Contribution:**

The findings and recommendations of the study could improve partogram utilisation in maternity care.

## Introduction

A partogram is a printed chart on which observations in labour are recorded in a graphic format to provide an overview of labour (Lavender, Hart & Smyth 2013:3). The partogram was initially developed by Friedman in 1954, based on the observations of cervical dilatation and foetal station against time elapsed in hours from the onset of labour (Lavender et al. 2013:3). Later in the same year, Friedman developed a cervicography showing four phases of cervical dilatation which were latent, acceleration, maximum slope and deceleration phases. According to Lavender et al. (2013:13), the cervicograph was used until 1972 when Friedman, Philpott and Castle developed the partogram into a tool for monitoring labour by introducing the action and alert lines to the graphical record. Furthermore, it was highlighted that an alert line represents the slowest 10% of primigravidae women’s labour progress, while an action line is placed 2 h after the alert line to prompt effective management of slow labour progress. The use of a partogram aims at alerting midwives and obstetricians about deviations in the progress of labour as well as maternal and foetal well-being (Orhue, Aziken & Osemwenkha 2012:1).

The World Health Organization (WHO 2020:1) recently reviewed and revised the design of the partogram to develop the Labour Care Guide (LCG). According to the WHO (2020:1), the LCG was designed for intrapartum monitoring of the well-being of women and foetus through regular assessments to identify any deviation from normal. The WHO recommends the universal utilisation of the partogram or LCG for all births in health facilities, including primary, secondary and tertiary care settings (WHO 2020:3). Routine use of partogram helps make better decisions for the diagnosis, prevention and management of prolonged and obstructed labour and recognise cephalopelvic disproportion (CPD). The WHO further confirms that routine partogram use during labour assists in a prompt decision on the transfer of a woman from one level of care to another and informs a practitioner whether to augment or terminate labour. The use of a partogram is a strategy to reduce intrapartum complications and reduce maternal and foetal/neonatal morbidity and mortality. Mukisa et al. (2019:2) confirmed that the appropriate and correct utilisation of the partogram can reduce complications from prolonged labour for the mother, such as obstetric fistula, postpartum haemorrhage, sepsis, uterine rupture, infant death, anoxia and infections.

The National Department of Health (DoH) of South Africa adopted the first WHO composite partogram that commences the active phase of labour at 4 cm cervical dilatation. According to the updated guideline for intrapartum care in South Africa, the former partogram was revised in March 2018 to commence the active phase of labour at 5 cm cervical dilatation and is still being piloted in some provinces. The DoH has adapted all seven sections from the previous composite partogram and applied them to the interim partogram (DoH 2016:41, 2019:4). This tool increases the quality and regularity of all observations on the foetus and the mother in labour, thus aiding early recognition of problems in either party. However, the proportion of healthcare workers and facilities consistently using the partogram is inadequate and likely to contribute to maternal and/or perinatal morbidity and mortality (DoH 2014:9).

According to the DoH (2014:1) in the Saving Mothers Report, the North West Province is one of the provinces with maternal mortality rates far above the national average, with the selected district having the highest at 310 per 100 000 live births. The selected district was one of the 10 districts with the most maternal deaths due to obstetric haemorrhage, 15% higher than the national average (DoH 2014:9). Obstetric haemorrhage is linked to prolonged labour, and partogram use can prevent primary postpartum haemorrhage, a complication caused by uterine atony because of prolonged labour (Dippenaar & Da Serra 2018:491).

A partogram is divided into sections for the recording of different characteristics:

- The progress of labour, which is monitored by measuring cervical dilatation, the descent of the head and uterine contractions.
- The foetal condition, which is monitored by assessment of foetal heart rate (FHR), the colour of amniotic fluid if membranes have ruptured, and moulding of the foetal skull.
- The maternal condition, which is assessed by monitoring pulse, blood pressure (Bp), temperature and urinalysis.

There is also a separate section to enter drugs administered like oxytocin, pain medication and intravenous fluids (DoH 2015:15). Failure to complete all activities and record them on the partogram constitute substandard care and a missed opportunity because deviations from the norm are not identified in time to initiate intervention early if necessary. During the active phase of normal labour, cervical dilatation should be plotted to the left of or on the alert line, and it should not move to the right of the alert line as labour may be prolonged.

Midwives and other healthcare providers must be competent in correct partogram recording and interpretation to manage labour and childbirth in order to achieve Sustainable Development Goal (SDG) 3. In a study conducted in Bangladesh, it was revealed that midwives and other healthcare workers only used a partograph to document and not as a guiding tool to monitor labour, foetal and maternal conditions (Khan et al. 2018:15). Similarly, in a study conducted in India, only 48.7% of the intrapartum monitoring section of the partogram was used indicating a knowledge gap of midwives rendering care (Palo et al. 2019:2687). This is consistent with the study conducted in Kenya, which revealed poor partogram utilisation and an exceptionally low proportion of completed partographs covering all three parameters: monitoring of the foetal condition, the progress of labour, and maternal condition (Mukisa et al. 2019:107). Bedwell et al. (2017:5) conducted a systematic review to determine when and how a partogram works and confirmed that overall completion of the partograph was poor compared to a predefined standard, thus impacting the tool’s utility in clinical practice. The researchers further concluded that the partograph sections most likely to be completed were those relating to the progress of labour and FHR. The section on maternal well-being was poorly plotted or was not completed at all.

According to Hailu et al. (2018:2), poor partogram utilisation in Ethiopia was in the form of failure to complete all parameters as expected but focusing only on the FHR and cervical dilatation. Researchers further confirmed that uterine contractions and vital signs were not considered, thereby missing out on vital information that could assist in achieving SDG 3 by a reduction in maternal and perinatal morbidity and mortality. Similarly, Melese et al. (2020:4) also reported a low percentage of utilisation of partograms for all labouring women. Partogram utilisation is a global problem based on the many studies conducted.

A study conducted in a private hospital in Gauteng Province revealed that midwives had knowledge of plotting correctly but not implemented and only one doctor was able to plot the partogram correctly (Yazbek & Jomeen 2020:05). This explains why women are subjected to unnecessary caesarean section as a mode of delivery with no clinical indication due to knowledge deficit. Midwives and other healthcare providers working in maternity units were concerned about empowerment and refresher courses on using the partogram to provide quality maternal and neonatal care.

There is a need for proper training of obstetric and midwifery caregivers on partogram utilisation to improve obstetric care and utilisation of this tool as recommended by Hailu et al. (2018:4). According to Ayehubizu et al. (2022:12), the following factors were found to be contributing to poor partogram utilisation: a lack of expertise and understanding, insufficient partogram training and a negative attitude displayed by healthcare providers. These findings coincide with the findings of Bazirete (2014:56) confirming that a significant percentage of midwives had a fair knowledge of the partogram and its utilisation in the management of labour, but despite that, a large portion of midwives complete the partogram poorly. Similarly, Opoku and Nguah (2015:6) highlighted that all birth attendants needed more education on the importance and correct usage of the partogram to ensure that all labouring women are monitored using this tool. Researchers further revealed that almost half of the labouring women were not monitored with the partogram because of a lack of knowledge regarding plotting and interpretation. Similarly, Konlan et al. (2016:3) revealed that most healthcare workers could not identify the specific time to commence recording the partogram and did not know the symbol of an X to be used for cervical dilatation as on the LCG as set by the WHO (2020:4). This is confirmed by Maphashaa et al. (2017:84) who found that healthcare workers were aware and knowledgeable of the partogram and its importance in the management of prolonged and obstructed labour, but did not know the symbols to be used when plotting on the partogram partogram (e.g. Fetal Heart Rate (FHR), Meconium Stained Liquor (MSL), and moulding). Lack of knowledge harmed their ability to complete partograms during labour and interpret abnormal findings.

Further research revealed that midwives had a negative attitude towards plotting a partogram because it was time-consuming and thought they needed more training on how to plot (Githae, Mbisi & Boraya 2019:12). According to Nyiawung et al. (2018:076), the prevalence of the use of the partogram was low in Cameroon because healthcare providers lacked training and mentorship on partogram use. Midwives were not getting support from their authorities on partogram utilisation. Managers in charge of maternal and neonatal care should ensure that training on the use and interpretation of a partogram takes place, as this is required in caring for women during labour and childbirth. Continuous and mandatory refresher courses, as well as hands-on workshops on the use of the partogram, according to Okusanya et al. (2018:53), will likely sustain good knowledge and improve the utilisation of partogram during labour and childbirth to achieve SDG 3. Similarly, Brits et al. (2020:6) found a need for regular training and monitoring of completed partogram as this could be a first step to improving the practice of partogram use by healthcare providers.

According to Ayenew and Zewdu (2020:9), obstetric care providers with adequate knowledge of partographs were 2.5 times more likely to utilise partographs than their counterparts. Furthermore, the same authors explain that the rationale for proper partogram utilisation could be that knowledge enables them to understand when there is a deviation from the normal progress of labour and allow a referral to another level of care to prevent unnecessary interventions such as caesarean sections. Furthermore, the researchers established that if obstetric care providers are provided with partogram refresher training, practical demonstration and supportive supervision, their effectiveness on partogram utilisation will be enhanced.

The objectives of this study were to assess the frequency of recording admission information and partogram parameters during the first and the second stages of labour and to describe the use of partograms in selected healthcare facilities in the district.

## Research setting

The district is in the northeast of the North West Province and consists of five subdistricts with an estimated population of 1 848 133. The study was conducted in four out of five subdistricts, five facilities providing low-risk maternity care and three providing all-risk maternity care, operating for 24 h. The fifth subdistrict was excluded from the study as it did not have a 24-h healthcare facility rendering maternity services and referred all maternity cases to Gauteng Province. The study was conducted in private rooms at each of the selected healthcare facilities.

### Study population

Population refers to the group of elements, which could be individuals, objects, events or substances with specific characteristics that are the focus of the study (Gray & Grove 2021:818). Elements under investigation in this study were plotted partograms of all selected healthcare facilities in the district within the past 6 months of data collection. The researcher wanted to assess and describe the recent practice of healthcare providers and identify gaps regarding partogram utilisation.

#### Inclusion criteria

The inclusion criteria were partograms of all women who:

- reported for labour with cervical dilatation on the admission of 0–5 cm
- delivered normally at a gestational period of 37 weeks and more
- were monitored during labour and later delivered by caesarean section
- delivered 6 months before data collection.

#### Exclusion criteria

The exclusion criteria were partograms of all women who:

- appeared in the delivery register but delivered in transit, either in ambulances or private vehicles, and at home because nothing about their labour process was documented
- appeared in the register admitted with cervical dilatation > 5 cm, booked for elective caesarean section, premature labour, placenta praevia and placental abruption.

### Sampling

A purposive sampling was used to select healthcare facilities rendering 24-h maternity services. Simple random sampling was used to select plotted partograms because each document had an equal and independent chance of being drawn and included in the study. A list of all plotted partograms was obtained, and a sampling frame was created. A fishbowl technique was used to randomly select a sample from each healthcare facility to ensure equal opportunity for each element to be selected.

### Sample size

The sample size was determined using Raosoft sample size calculator available at http://www.raosoft.com. The size was estimated using the single proportion formula by assuming a 5% marginal error with a confidence level of 95% (=0.05). The total population was 908, and the total sample was 279.

### Data collection

Data were collected in labour wards of the selected healthcare facilities from 01 September 2013 to 30 September 2013 from 07:30 to 17:00 with breaks in between. The researcher assessed partograms of deliveries conducted from 01 March 2013 to 01 September 2013 and used maternity registers to confirm if deliveries occurred at that facility. The researcher believes that although data were collected more than 5 years ago, the findings of this study are still relevant to the existing problems on partogram utilisation.

Data were collected using a checklist developed by the researcher. The checklist included all the patients’ information that appeared on the partogram except names. The researcher assessed all parameters that should be logged on admission, namely, the patients’ name, parity, gravida, pelvic assessment, whether the pelvis was adequate or not, duration of labour, duration of rupture of membranes and risk factors, FHR, the colour of the liquor, caput, and moulding, blood pressure and pulse rate, temperature, urinalysis and use of syntocinon or other drugs where applicable. A colleague assisted the researcher in checking the comprehensiveness of all checklists to ensure that all information had been correctly collected and recorded. During the processing of data, the researcher and the colleague rechecked whether the information was complete or not to ensure consistency. The operational manager assigned ward clerks to retrieve patients’ records from the filing room and those still in the ward.

During auditing, a tool number was assigned to each partogram and indicated whether the item was recorded (1), not recorded (0) or not applicable by using a tick. If the recording was incomplete, it was ticked as not recorded.

Criteria items per section were determined as the number of recorded items divided by the total number of items equal to the score obtained. A score of 69% or less implies poor utilisation and 70% or more implies optimal utilisation.

The researcher assessed the progress of labour, focusing on the descent of the foetal head in relation to the pelvic brim, the level of the head in relation to the ischial spines, cervical dilatation, effacement and uterine contractions. The recording of medication given and if the management plan was recorded were also checked. The second stage of labour and the mode of delivery were assessed if it was recorded or not. The mode of delivery was also assessed if it was a normal vaginal delivery, assisted ventouse or forceps delivery, or caesarean section. Neonatal and maternal outcomes were assessed whether good or adverse and whether recorded or not recorded and recording of outcomes.

A pilot study was conducted on five files that met the inclusion criteria to investigate the feasibility of the study and to detect the possible flaws with the methodology and the data collection tool. The methodology was modified to include sampling in the choice of clinical facilities and the checklist was updated with more details to identify poor partogram utilisation and optimal utilisation.

### Data analysis

Raw data from the checklist were captured into a Microsoft Excel spreadsheet, checked for correctness and completeness by the researcher, and verified by the supervisor. Data were analysed using Statistical Package for Social Sciences (SPSS) software version 22. Frequency distributions and percentages were used in the description of partogram utilisation.

### Ethical considerations

The research proposal was presented to the North-West University Health Research Ethics Committee and obtained ethical clearance numbered NWU-00053-13-A9. Approval was also obtained from the North West Department of Health, and permission was obtained from chief executive officers of hospitals and subdistrict managers of all community health centres/midwife obstetric units.

Confidentiality and anonymity were ensured by identifying clinical facilities under investigation as healthcare facilities ‘A, B, C, D, E, F, G and H’. As data were collected, the names of participants were not recorded on the tool to safeguard the anonymity of the owners of the files. After data were collected in a private room, completed checklists were stored in a locked cupboard that could only be accessed by the researcher. There were no human participants in this study.

## Results

Data from the partograms of 279 women who delivered and who met the inclusion criteria were collected with checklists. The names of participants were not recorded on the checklist. Some results are presented in Table 1, Table 2 and Table 3.

**TABLE 1:** Patients’ information to be recorded on admission.

Patients’ information	*n*	% recorded	*n*	% unrecorded
Names	234	83.9	45	16.1
Parity	252	90.3	27	9.7
Gravida	238	85	41	15
Pelvis adequacy	152	54.5	127	45.5
Duration of labour	135	48.4	144	51.6
State of membranes and liquor	145	54	134	48

*Source:* Mabasa, S.K.M., 2018, ‘Evaluation of partogram utilization in maternity care in selected health care facilities of Bojanala District. Mafikeng: North-West University, Mafikeng Campus’, (Dissertation - Master of Nursing Science in Community Nursing (Midwifery and Neonatal Nursing Science)

**TABLE 2:** Partogram parameters during the latent phase of labour.

Partogram parameters	*n*	% recorded	*n*	% unrecorded
**Foetal conditions**				
Foetal heart rate (FHR)	96	34.4	183	65.6
Foetal heart rate pattern	20	7.2	259	92.8
Liquor status	62	22.2	217	77.9
Presence/absence of caput	59	21.1	220	78.8
Presence/absence of moulding	64	22.9	215	77.1
Position of the head	13	4.7	266	95.3
**Progress of labour**				
Cervical dilatation	86	30.9	193	69.2
Cervical effacement	21	7.5	258	93.1
Station of the head	52	18.6	227	81.4
Head above the brim	10	3.6	269	96.0
Uterine contractions	59	21.1	220	78.8
**Maternal conditions**				
BP and pulse	26	9.3	253	90.7
Temperature	27	9.7	252	90.4
Urinalysis	23	8.2	256	91.8
Medication such as pain relief, antibiotics, and intravenous fluids	27	9.7	252	90.4
**Management plan**				
Further management plan	27	9.7	252	90.4

*Source:* Mabasa, S.K.M., 2018, ‘Evaluation of partogram utilization in maternity care in selected health care facilities of Bojanala District. Mafikeng: North-West University, Mafikeng Campus’, (Dissertation - Master of Nursing Science in Community Nursing (Midwifery and Neonatal Nursing Science)

**TABLE 3:** Partogram parameters during the active phase of labour.

Partogram parameters	*n*	% recorded	*n*	% unrecorded
**Foetal conditions**				
Foetal heart rate (FHR)	154	55.2	125	44.8
Foetal heart rate pattern	37	13.3	242	86.7
Liquor status	69	24.7	210	75.3
Presence/absence of caput	88	31.5	191	68.5
Presence/absence of moulding	85	30.5	194	69.5
Position of the head	29	10.4	29	10.4
**Progress of labour**				
Cervical dilatation	129	46.2	150	53.8
Cervical effacement	24	8.6	56	91.4
Station of the head	60	21.5	219	78.5
Head above the brim	22	7.9	257	92.1
Uterine contractions	83	29.7	196	69.9
**Maternal conditions**				
BP and pulse	42	15.1	237	84.9
Temperature	34	12.2	245	87.8
Urinalysis	20	7.2	259	92.8
Medication such as pain relief, antibiotics, and intravenous fluids	15	4.7	264	95.3
**Management plan**				
Further management plan	16	5.7	263	94.3

*Source:* Mabasa, S.K.M., 2018, ‘Evaluation of partogram utilization in maternity care in selected health care facilities of Bojanala District. Mafikeng: North-West University, Mafikeng Campus’, (Dissertation - Master of Nursing Science in Community Nursing (Midwifery and Neonatal Nursing Science)

### The second stage of labour

The second stage begins from the time the cervix is fully or 10 cm dilated up to the time the foetus is ultimately expelled from the birth canal (DoH 2015:49). Knowledge of the commencement of the second stage of labour assists in diagnosing the prolonged second stage of labour which can cause postpartum haemorrhage and birth asphyxia. In this study, 69 (24.7%) partograms had the second stage of labour recorded, and 210 (75.2%) partograms had no recording.

### Mode of delivery

The mode of delivery was recorded on 196 (70.3%) partograms and 83 (29.7%) were not recorded. Of those recorded, 182 (92.8%) were spontaneous vaginal deliveries, 14 (7.1%) were assisted deliveries by ventouse, and 83 (29.7%) were deliveries by caesarean sections.

### Maternal outcomes

Maternal outcomes were recorded on only 163 (58.4%) of the partograms, and 116 (41.6%) were not recorded. Out of 279 partograms assessed, the following adverse maternal outcomes were identified from patient files, and some were only recorded in the delivery register: 89 (32%) had a primary postpartum haemorrhage, 22 (7,9%) had retained placenta, 47 (17%) had third-degree perineal tears and 12 (4.3) had cervical tears.

### Neonatal outcomes

Neonatal outcomes were recorded on 139 (49.8%) of the partograms, and 140 (50.2%) were not recorded. The following adverse neonatal outcomes were identified: 20 (7.1%) had respiratory distress at all levels of care, 15 (5.4%) had meconium aspiration, 8 (2.9%) had transient tachypnoea, 40 (14.3%) had severe birth asphyxia, 33 (11.8%) had low Apgar score, 25 (8.9%) were fresh stillbirths and 10 (3.5%) were early neonatal deaths.

## Discussion

It is mandatory in South Africa that all women in labour should be monitored using a partogram and that all findings should be recorded on admission (DoH 2016:41). There was no shortage of partograms in all facilities under investigation as revealed by its availability in all files. The findings contrast with those of Maphasha et al. (2017:84), who found that partograms were not available in the unit in some files.

In this current study, a significant percentage of partograms had the names of patients recorded, while 45 women were in labour without their names being recorded on the partogram. Furthermore, a very high percentage of partograms had no parity and gravida indicated. Knowledge of women’s parity and gravida helps midwives and doctors be on the alert when managing women in labour to identify possible risk factors to intervene appropriately to guide the management. Women who have given birth five times or more are at risk of pregnancy-labour-related complications leading to morbidity and/or mortality (Dippenaar & Da Serra 2018:242).

Routine and full pelvic assessment are not recommended, but this may be done as part of labour care if the presenting part has not engaged. Midwives and other healthcare providers should indicate if the pelvis is adequate or not. In this study, a significant percentage of partograms had no recording of the state of the pelvis and very few partograms were recorded. The findings of this study revealed that the duration of labour was recorded on a few partograms, and many were not recorded as presented in the results. Information on the duration of rupture and non-rupture of membranes was not recorded on a significant percentage of partograms. It is vital to record the duration of the rupture of membranes as these assist in diagnosing prolonged rupture of membranes, which places both the mother and the foetus in utero at risk of infection. The study also revealed that the recording of risk factors was only indicated in a few partograms and many were not recorded. Each woman must be risk-classified to determine the level of care at which she should be managed (DoH 2019:11).

During the latent phase, the foetal conditions, maternal conditions and the progress of labour should be recorded on the partogram provided that a correct diagnosis of labour is made. Findings revealed a very low percentage of recorded partograms, and a significant percentage of partograms were not recorded. During the active phase, foetal conditions, the progress of labour and maternal conditions should be recorded on the partogram. The monitoring of the foetal condition during labour includes FHR, absence or presence of decelerations, and variability. Foetal heart rate should be recorded half hourly in the first stage of labour (DoH 2019:11; WHO 2020:12). In this current study, during both the latent and active phases of labour, the recording of FHR and FHR pattern revealed a poor recording of these parameters. Evidence by Adesola et al. (2014:682) in a study conducted in Ile-Ife, Nigeria, established that the FHR was incorrectly or not charted in over 50% of the case files. The baseline of the FHR and the FHR pattern were not recorded according to the standard, and based on this there was a high probability of having missed diagnosing foetal distress in time. Similarly, Brits et al. (2020:6) reported that healthcare workers could not identify foetal distress as the FHR and pattern were not plotted; as a result, the foetal condition was not known. The inability to chart FHR per protocol has been associated with an increased intrapartum foetal death (Okusanya et al. 2018:52). A significant percentage of 54% of the condition of amniotic fluid, presence or absence of caput, absence and/or presence of moulding, and deceleration was not plotted. Similarly, Maphasha et al. (2017:84) confirmed that healthcare workers could not plot moulding, caput and FHR. This is consistent with the study conducted by Chemeda, Teklewold and Daka (2019:36) with low standard recordings in mouldings (2%), liquor status (3.2%) and descent (3.9%). The foetal brain is protected from compression if the moulding is not increasing or excessive. According to Dippenaar and Da Serra (2018:406), if there is excessive or increasing moulding when the foetal head is high in the pelvis, it warns healthcare providers of the possibility of CPD and obstructed labour. The degree of moulding should be assessed four hourly and recorded to enable midwives and other healthcare workers to diagnose CPD and obstructed labour and intervene promptly.

The progress of labour was only recorded with the initial assessment. This practice is of concern as the latent phase of labour is not considered until when the woman complains of severe pain and problems are not identified if the woman is not assessed at least every 4 h. Labouring women were subjected to a prolonged latent phase of labour, where 65% of plotted partograms were seen when women were in labour for more than 24 h. The progress of labour was also poorly recorded during the active phase of labour with a high percentage of recorded cervical dilatation, effacement, and station of the presenting part and extremely high unrecorded parameters. Similarly, a study conducted in Nigeria by Okusanya et al. (2018:52) confirmed a significant percentage of unplotted uterine contractions during both the latent and active phases of labour. The study also revealed a substandard recording of uterine contractions or none at all. This makes it impossible to recognise deviations from the average on the progress of labour and to identify if labour was progressing well or not for necessary interventions to be taken. Findings were consistent with Okusanya et al. (2018:52), where it was established that although a partogram was utilised, it was incompletely filled with an inadequate and substandard recording of uterine contraction specifically during the active phase of labour. In this study, it was revealed that midwives and other healthcare workers lacked knowledge on plotting cervical effacement and station of the presenting part. Maphasha et al. (2017:84) also confirmed that healthcare professionals were incompetent in plotting parameters as expected because of a lack of knowledge. A significant percentage of 55% of no recordings were seen on the station of the presenting part above the ischial spines, the level of the head to the pelvic brim, and uterine contractions. Some parameters were completed selectively or not at all in this study at all levels of care.

This study revealed that the maternal condition was the most ignored part compared to the foetal condition and the progress of labour in most partograms. Maternal vital signs were recorded in the initial assessment in all partograms, but the recording was minimal during the subsequent assessments. All vital signs were poorly recorded in a high percentage of partograms, blood pressure and pulse, temperature and urinalysis. All vital signs are to be recorded so as not to miss problems like infections with elevated temperature, elevated blood pressure and urine abnormalities to exclude preeclampsia or other medical conditions that may have adverse effects on the mother and the foetus. Poor and lack of recording especially blood pressure and urinalysis are of great concern as hypertensive disorders of pregnancy, especially, preeclampsia may be undiagnosed in labour until women develop eclampsia. Okusanya et al. (2018:55) and Chemeda et al. (2019:42) confirmed that maternal temperatures and urine output were parameters that were not recorded at all during all phases of labour. The bladder should be emptied, and urine tested for glucose and ketones, as a full bladder can interfere with the progress of labour and ketosis is a sign of maternal starvation. Similarly, Jere (2014:24) revealed that urine output was documented in 7.4% (12/162) of the partograms. This was also confirmed by Zelellw and Tegegne (2018:7) that out of 147 women who delivered, only one delivering mother’s urine was checked for its volume, protein and ketone bodies during labour. Jere (2014:24) found that temperature was documented entirely in only 11% (18/162), and pulse rate and blood pressure in only 4.9% (8/162) partograms of women during both the latent and active phases of labour. The woman’s temperature should remain normal and be checked and recorded every 4 h because an elevated temperature indicates infection, especially in the presence of premature rupture of membranes, ketosis or maternal dehydration. The woman’s pulse should also be normal and be recorded accordingly because tachycardia is associated with anxiety, infection, pain and reaction to medication, and recording of this should not be missed. This implied that a significant percentage of women during the latent, active phases of labour and subsequent management had poor vital signs monitoring.

Recording of medication given during labour, such as pain relief, antibiotics where necessary and intravenous fluids, was poor, and a high percentage was not recorded. The management plan on all partograms for both the latent and the active phases of labour was poorly documented and indicated a high percentage of unrecorded partograms. All medication and fluids administered should be recorded as well as identified problems and the management plan (DoH 2014:41). Administration of pain relief to women in labour is part of supportive woman-centred care that needs to be implemented by healthcare workers as it shortens labour and promotes a positive birth experience. If it is not recorded, it is assumed not to be done.

There were no recordings of the second stage of labour, and it could be that many women delivered by caesarean section without any indication. Of the partograms assessed, 29.7% had a caesarean section performed; no indication was discovered for this procedure. The study found most women did not receive many observations during this stage of labour. Problems such as a prolonged second stage of labour could have been missed, affecting maternal and neonatal outcomes. The mode of deliveries was recorded in a high percentage of partograms. In obstetrics and midwifery practice, the history of previous deliveries and the reason for undergoing such a delivery method are crucial as they help in the decision-making of the mode of delivery for the current pregnancy. The study showed a significant percentage of caesarean section rates as compared to the norm of 11%. Similarly, the study by Jere (2014:27) confirmed that most women (57.8%) delivered spontaneously, but 37.5% delivered by caesarean section with no indication. This confirms the importance of recording in the partogram to avoid subjecting the woman to an inappropriate mode of delivery.

There were several partograms with maternal outcomes recorded, but there were also a sizeable number that were not recorded. Adverse maternal outcomes in this study were primary postpartum haemorrhage (PPH), retained placenta, and second- and third-degree perineal and cervical tears. Similarly, Jere (2014:39) revealed the same adverse maternal outcomes being caesarean section deliveries, PPH, genital infection and perineal trauma, though without any significant statistical differences. Chemeda et al. (2019:39) found that relatively few mothers experienced adverse outcomes, such as PPH or third-degree genital tears, and a very small proportion received a blood transfusion.

One hundred and forty partograms (50.2%) had no recorded neonatal outcomes, compared to 139 (49.8%) with recorded outcomes. Adverse neonatal outcomes in this study refer to respiratory distress at all levels of care, low Apgar score, meconium aspiration, transient tachypnoea, severe birth asphyxia, fresh stillbirths and early neonatal death. Similarly, Jere (2014:39) reported adverse neonatal outcomes of low Apgar scores and fresh stillbirths. These results are consistent with the research conducted by Rani et al. (2016:316) and Chemeda et al. (2019:36), which discovered that a noteworthy proportion of newborns were small for gestational age, had septicaemia, neonatal jaundice, low Apgar score and birth asphyxia. Appropriate application of the partogram allows for the diagnosis of prolonged and obstructed labour and decreases infant problems such as anoxia, infections, mortality and stillbirth risks (Dippenaar & Da Serra 2018:399).

The overall partogram utilisation in this study indicated that 20% were correctly completed, while 80% of substandard and incomplete utilisation, and no recording at all. Poor utilisation in this study could be attributed to a shortage of midwives and midwife specialists in most healthcare facilities under investigation. Madaha and Mabenge (2021:13) and Nyiawung et al. (2018:080), and Abuga (2020:56) confirmed that staff shortage, increased workload in maternity units, high patient turnaround time in the labour ward, healthcare providers’ negative attitudes towards the partogram, and poor knowledge of plotting the partogram could be some of the reasons for the high incorrect partogram utilisation. Jere (2014:33); Markos, Arba & Paulos (2020:5), and Madaha & Mabenge (2021:13) also confirmed that although partograms were used, they were poorly used by health providers despite their availability in women’s files. This was also confirmed by Maphasha et al. (2017:84) who stated that despite evidence of safe and successful labour using the partogram, this tool remains inconsistently and incorrectly utilised. This is consistent with the studies conducted in Nigeria where suboptimal knowledge and poor utilisation of partogram among primary healthcare providers were found coupled with skill incompetency, lack of motivation, negligence and a shortage of resources (Ango et al. 2020:73; Zelellw & Tegegne 2018:13). Studies by Ayenew and Zewdu (2020:6) revealed that overall prevalence of partogram utilisation among obstetric care providers was low.

No partogram reviewed in the current study was completed according to the standard. This is a confirmation that healthcare providers in maternity units need more knowledge on partogram utilisation. Few studies confirmed that healthcare providers in maternity units had optimal knowledge about partograms but poor utilisation (Zelellw & Tegegne 2018:13). According to Gebreslassie et al. (2019:4), the provision of on-the-job training on the partogram is recommended to improve the knowledge and attitude of obstetric care providers towards the partogram utilisation. During the data collection process, the researcher observed that in most selected healthcare facilities across all subdistricts, one or two midwives were monitoring three to four labouring women. This may have contributed to the inadequate and poor use of partograms.

## Strengths and limitations

Elements under investigation in this study were plotted partograms of women who delivered. It was easy to obtain information as data were already available in the maternity case records. Data were collected within 4 weeks in four out of five subdistricts of the district. The findings of the study can still be generalised to other districts in the North West Province with similar contexts. Although some files that met the inclusion criteria were allegedly kept in the manager’s office, it was possible to address the objectives of the study, recordings of partogram parameters during the second stage of labour, and overall utilisation of the partogram in the district. This study was carried out successfully and has produced significant results.

The researcher was limited by reviewing records; if the study had included face-to-face interviews with midwives and other healthcare workers, more information could have been obtained which did not appear on the partograms. Some records of adverse neonatal and maternal outcomes were not available for review at most midwife obstetric units, as they were allegedly sent with patients to other levels of care. These challenges made the researchers evaluate the records that could be found. The researcher acknowledges the possibilities of in-service training on partogram utilisation that might have taken place, and the situation could have changed as this study happened years ago.

## Conclusion

The partogram is a mandatory tool to be used for intrapartum care in South Africa. It is a simple low-cost monitoring tool, and if used appropriately, it enables healthcare providers to detect abnormalities early and intervene appropriately. Recording on the partogram seemed to be directed at the progress of labour and the FHR with a limited focus on maternal well-being. Proper documentation of all parameters is vital even in low-risk pregnancies, so that complications may be detected as soon as they occur. Maina, Mutungwa-Muwenda and Karonjo (2016:82) revealed that the progress of labour was frequently documented, and maternal and foetal conditions were incompletely documented.

The study revealed that although partograms were available in all the files, none was 100% according to standards; only 20% met some standards as determined by the WHO (DoH 2016:41) and 80% were substandard, with some not recorded at all. The study revealed poor partogram utilisation by healthcare workers in four out of five subdistricts of the district. The study found that there were inadequate clinical practices regarding partogram usage in some labour wards of healthcare facilities under investigation within the district. Labour parameters had been recorded at least once in the partogram during all phases of labour with little and no information at all during the latent phase of labour. Some relevant data were missing due to inconsistency and incomplete documentation in the patient’s files, or no documentation.
